# Free-Ranging Pig and Wild Boar Interactions in an Endemic Area of African Swine Fever

**DOI:** 10.3389/fvets.2019.00376

**Published:** 2019-10-30

**Authors:** Estefanía Cadenas-Fernández, Jose M. Sánchez-Vizcaíno, Antonio Pintore, Daniele Denurra, Marcella Cherchi, Cristina Jurado, Joaquín Vicente, Jose A. Barasona

**Affiliations:** ^1^VISAVET Health Surveillance Centre, Animal Health Department, Faculty of Veterinary, Complutense University of Madrid, Madrid, Spain; ^2^Istituto Zooprofilattico Sperimentale della Sardegna, Sardinia, Italy; ^3^Spanish Wildlife Research Institute (IREC) (CSIC-UCLM), Ciudad Real, Spain

**Keywords:** free-ranging pig, wild boar, camera trapping, interactions, critical time window, African swine fever

## Abstract

African swine fever virus (ASFV) is spreading throughout Eurasia and there is no vaccine nor treatment available, so the control is based on the implementation of strict sanitary measures. These measures include depopulation of infected and in-contact animals and export restrictions, which can lead to important economic losses, making currently African swine fever (ASF) the greatest threat to the global swine industry. ASF has been endemic on the island of Sardinia since 1978, the longest persistence of anywhere in Eurasia. In Sardinia, eradication programs have failed, in large part due to the lack of farm professionalism, the high density of wild boar and the presence of non-registered domestic pigs (free-ranging pigs). In order to clarify how the virus is transmitted from domestic to wild swine, we examined the interaction between free-ranging pigs and wild boar in an ASF-endemic area of Sardinia. To this end, a field study was carried out on direct and indirect interactions, using monitoring by camera trapping in different areas and risk points. Critical time windows (CTWs) for the virus to survive in the environment (long window) and remain infectious (short window) were estimated, and based on these, the number of indirect interactions were determined. Free-ranging pigs indirectly interacted often with wild boar (long window = 6.47 interactions/day, short window = 1.31 interactions/day) and these interactions (long window) were mainly at water sources. They also directly interacted 0.37 times per day, especially between 14:00 and 21:00 h, which is much higher than for other interspecific interactions observed in Mediterranean scenarios. The highly frequent interactions at this interspecific interface may help explain the more than four-decade-long endemicity of ASF on the island. Supporting that free-ranging pigs can act as a bridge to transmit ASFV between wild boar and registered domestic pigs. This study contributes broadly to improving the knowledge on the estimation of frequencies of direct and indirect interactions between wild and free-ranging domestic swine. As well as supporting the importance of the analysis of interspecific interactions in shared infectious diseases, especially for guiding disease management. Finally, this work illustrates the power of the camera-trapping method for analyzing interspecific interfaces.

## Introduction

African swine fever (ASF) is a viral disease of swine, affecting both domestic pigs, and wild boar (*Sus scrofa*) of all ages and sexes (1). There is no vaccine nor treatment available to fight ASF. Therefore, the control strategy is based on the implementation of strict sanitary measures (2, 3). These measures include depopulation of infected and in-contact susceptible animals, based on the specific contingency plans for ASF of each affected country, and export restrictions, which can lead to important economic losses. These devastating economic consequences suffered by affected countries along with the unprecedented spread through Eurasia since 2007 (4, 5), make ASF the current greatest concern to the global swine industry. ASF was first detected outside Africa in 1957 on the Iberian Peninsula, from where the virus spread throughout many other countries in Europe and Central and South America. These outbreaks have been effectively controlled except in Sardinia (Italy) (6), where the disease has remained endemic since 1978 (7).

The four-decade endemicity of ASF in Sardinia has led to substantial efforts to identify factors responsible for the failure of eradication programs of the island (7–12). When ASF was endemic on the Iberian Peninsula, the presence of soft ticks of the genus *Ornithodoros* (*O. erraticus*) proved to be one of the greatest challenges in controlling the spread (6). However, *Ornithodoros* ticks are not present in Sardinia (13). Instead, the likely endemic factors appear to be lack of farm professionalism including limited biosecurity conditions, high densities of wild boar in the area and local practices such as raising non-registered domestic pigs (free-ranging pigs) in communal lands (7–9, 11, 12, 14). Within these factors, several studies suggest that the most important is the presence of non-registered domestic pigs, which is related to socioeconomic, cultural and traditional aspects (7–9, 14–17).

These animals are domestic pigs bred under free-ranging conditions for their entire life span, although they are occasionally fed by their owners during winter and summer seasons, when food is scarce in the natural environment (18). This practice is strongly rooted in tradition because it costs little to feed the pigs and their meat can fetch high prices on the local market. Sardinian authorities forbade the practice of raising free-ranging pigs in 2012 (19), and this ban was reiterated in the latest ASF eradication plan (PE-ASF15-18; Regional Decree Number 5/6, 6 February 2015), which also called for rapid eradication of cases when they occurred on registered holdings and incentivized good swine breeding practices (20, 21). However, no information on the sanitary status of free-ranging pigs was available up to 2019, and it showed higher ASF prevalence in free-ranging pigs than in wild boar and registered domestic pigs (14).

Susceptible pigs in direct and indirect contact with infected wild boar with ASF virus (ASFV), strain Armenia08, became infectious (22). This suggests that ASFV can be transmitted via direct between wild boar and domestic pigs, but also by environmental contamination [indirect; (22)] Free-ranging pigs share habitat with wild boar and can serve as a virus reservoir in Sardinia that provides a route of transmission between domestic pigs kept in backyards and wild boar populations (14). In fact, a recent study identified the combination of estimated wild boar density and mean altitude above sea level as one of the most significant risk factors, and free-ranging pigs commonly inhabit in mountainous areas (8). These considerations support the hypothesis that interaction between free-ranging pigs and wild boar was substantial to maintain ASF in Sardinia, yet we are unaware of published analyses of these interactions. Studies in other contexts have shown that intra- and interspecific interactions are socially, spatially and temporally structured, and their variations can influence the magnitude of outbreaks and the endemicity of infectious diseases (23–28). Different approaches have been taken to study animal interactions, such as questionnaires (26, 29), direct observations (30, 31), and telemetry (24, 25, 28). Another method is camera trapping, which provides a non-invasive way to collect direct and visual evidence of interactions (23, 32–34).

The current study, based on camera trapping, provides perhaps the first detailed insights into the frequency of direct and indirect interactions between free-ranging pigs and wild boar in an ASF-endemic area. The results support the importance of direct and indirect interactions between wild and free-ranging domestic pigs in ASF endemicity.

## Materials and Methods

### Study Area

The study was carried out in two Sardinian provinces, Nuoro and Ogliastra, located in the central-east part of the island, where illegal breeding of free-ranging pigs is especially common (8, 15). This region has a Mediterranean climate with a mean temperature of 14°C year-round, 12.4°C in the spring, and 20.5°C in the summer (35). These provinces are traditionally considered the ASF-endemic region in Sardinia, because there the disease has persisted longer, and recent outbreaks have occurred more frequently, than elsewhere on the island (7, 11). The three ASFV hosts on the island coexist in this area: registered domestic pigs, free-ranging pigs, and wild boar.

Within this endemic area, we collected data at the border between these two provinces, in the National Park of the Bay of Orosei and Gennargentu (Supplementary Material 1), where data from the Istituto Zooprofilattico Sperimentale della Sardegna indicate high wild boar density (8). This area is wooded and mountainous, and it is surrounded by many pig holdings. More than 88% of these holdings contain fewer than 11 pigs and conduct non-professional pig production under limited biosecurity conditions (7).

### Camera Trapping

Camera trapping surveys were conducted with heat- and motion-triggered infrared cameras (Model Ltl−6210 M, Little Acorn Outdoors, Denmark, Wisconsin, USA) left in the field at 15 different sites between April and August 2014, during spring and summer, to continuously monitor the area and recording images of animals. This non-invasive method did not require ethical approval. The date and time of each exposure was recorded. Cameras were placed to cover water sources and pasture areas as likely sites of animal congregation.

Two researchers independently analyzed the camera images manually. The following data were entered in an Excel 2007 spreadsheet: camera identifier, date (dd/mm/yyyy), start time of each animal observation (h:min:sec), animal subspecies (free-ranging pig or wild boar), animal age class (piglet, young, adult) and animal activity (moving, drinking, pasturing, inspecting, resting, washing). The different activities carried out by the animals observed have a great interest from a sanitary point of view, since activities which differ from movement, such as drinking or resting, imply a higher risk of ASFV transmission. In this sense, if a pig had several different behaviors, we have considered the most risky activity (Washing > Drinking > Pasturing > Resting > Inspecting > Movement).

Data were logged for each individual animal observation in a visit, which was defined as one or more images of the same subspecies until consecutive images were captured at least 10 min apart. This interval cut-off was chosen because an earlier study with ear-tagged wild boar in two areas of England indicated that animals rarely returned to the same area within 10 min (36). For each visit, the maximum number of animals from the same subspecies simultaneously present in any of the images was recorded. Since animals were not individually tagged, we assumed that animals in separate visits were distinct.

### Interaction Rates

We wanted to define the risk of ASFV transmission associated with each visit. To do so, we defined critical time windows (CTWs) during which ASFV could remain viable in the environment and be transmitted to other animals. We reviewed the literature for ASFV survival and infectious times in the environment by searching Web of Knowledge and PubMed databases from 1980 to December 2018 using the following topic search terms: African swine fever virus AND environment AND (survival OR transmission OR inactivation).

**Figure 1:**
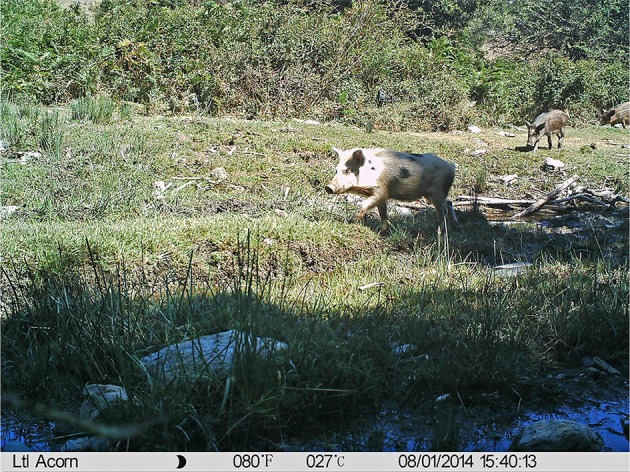
Example of a camera trapping image showing direct interaction between a free-ranging pig and wild boar.

**Figure 2:**
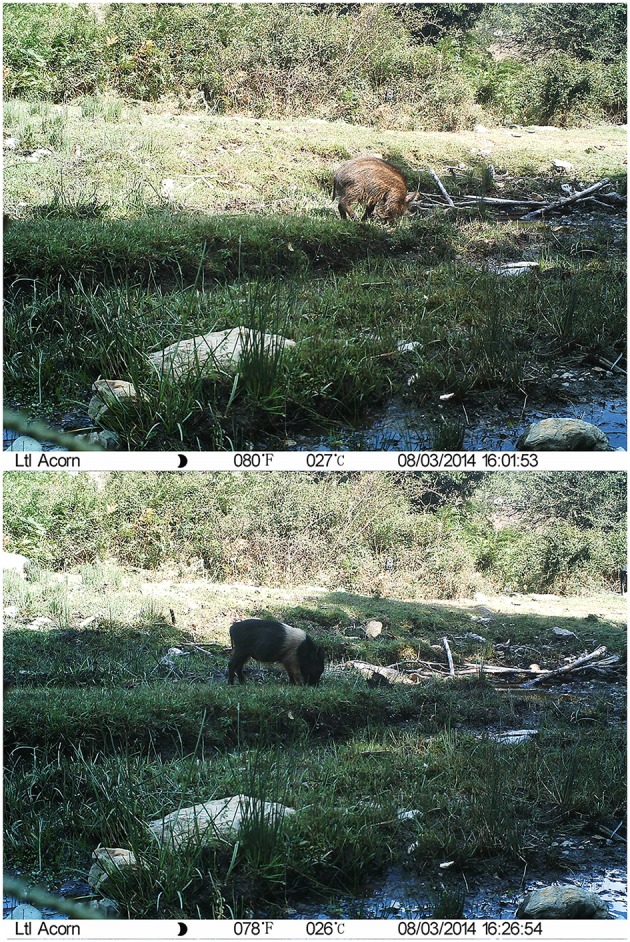
Example of a camera trapping image showing indirect interaction between a free-ranging pig and wild boar.

Direct interactions were defined as the simultaneous presence of free-ranging pigs and wild boar in the same image (Figure 1). Indirect interactions were defined as the presence of either free-ranging pigs or wild boar in one or more images, followed by the presence of the other subspecies within a specific CTW (Figure 2). Indirect interactions were determined based on the start date and time for each individual observation and counted using a MySQL database and PHP scripts (Supplementary Material 2).

### Data Analysis

Microsoft Excel 2013 and R 3.5.0 were used to analyze camera trapping data (37). Daily activity profiles were generated for free-ranging pigs and wild boar based on the proportion of animal observations that occurred in each hour of the day and in each season (23). Generalized linear mixed-effects models were conducted to identify factors influencing direct and indirect interaction rates. The models were specified with a negative binomial distribution because of the counting data and over dispersion (38).

The following potential predictors were considered because of their biological relevance for explaining free-ranging pig-wild boar interactions (Table 1). The categorical variables were the following: season, hour range (categories selected based on the observed daily activity profiles), direction of the interaction, age, animal activity, water source, and pastureland. The continuous variable was altitude. Direct interactions did not have a direction, so this variable was omitted from the model. In order to control the spatial correlation among observations, a variable identifying eight proximity area groups, from the 15 sites of camera trapping, was included in all models as a random factor.

**Table 1:** List of explanatory variables included in the generalized linear mixed model (negative binomial distribution and log link function) as risk potential factor for free-ranging pigs and wild boar interactions.

**Variable**	**Risk type**	**Categories**
Season	Temporal	Season 1: Spring
		Season 2: Summer
Hour range	Temporal	Hour range 1: 06:00–13:00 h
		Hour range 2: 14:00–21:00 h
		Hour range 3: 22:00–05:00 h
Direction of the interaction	Social	Direction 1: Wild boar followed by free-ranging pig
		Direction 2: Free-ranging pig followed by wild boar
Age class	Social	Age 1: Juvenile
		Age 2: Adult
Animal activity	Social	Activity 1: Moving
		Activity 2: Other activity
Water source	Environmental	Water source 1: Absence
		Water source 2: Presence
Pastureland	Environmental	Pastureland 1: Absence
		Pastureland 2: Presence
Altitude	Environmental	*Continuous variable*: 900–1,350 m

A data exploration followed by a backward stepwise model selection based on the Akaike information criterion was performed (39), and the Bayesian information criterion was also taken into account in order to obtain the most parsimonious model (40). The final generalized linear mixed-effects models for the negative binomial family were performed using the *glmer.nb* function from the R-package MASS (41). The overdispersion of residuals was checked by the sum squared Pearson residuals and the degrees of freedom. The differences associated with *p* < 0.05 were considered statistically significant.

## Results

During 375 trapping days, 434 observations of free-ranging pigs and 302 of wild boar were recorded (Table 2). Adult free-ranging pigs were more frequent than juveniles (chi-squared test, *p* < 0.01), whereas adult and juvenile wild boar were balanced. Observations of pigs and wild boar were significantly more frequent in summer than spring, and this seasonal difference was greater for wild boar (chi-squared test, *p* < 0.01).

**Table 2:** Observations of free-ranging pigs and wild boar, stratified by season, and age class.

		**Free-ranging pig**	**Wild boar**	**Total**
Season	Spring	162	67	229
	Summer	272	235	307
Age class	Juvenile	118	152	270
	Adult	316	150	466
Total		434	302	736

**Figure 3:**
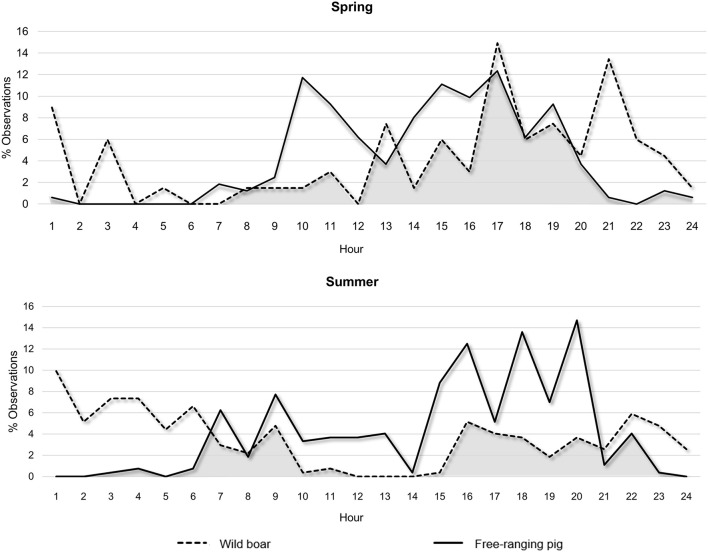
Daily activity profile of free-ranging pigs and wild boar, expressed as the percentage of total observations by hour of day and season (spring or summer). The overlap in the profiles for the two subspecies is represented in gray.

Free-ranging pigs were diurnal, showing a peak of activity between 15:00 and 20:00 h (Figure 3). Wild boar were mainly crepuscular/nocturnal, showing prolonged night-time activity. Some diurnal activity of wild boar was observed, which was more frequent in spring than summer.

### Direct Interaction Rate

**Figure 4:**
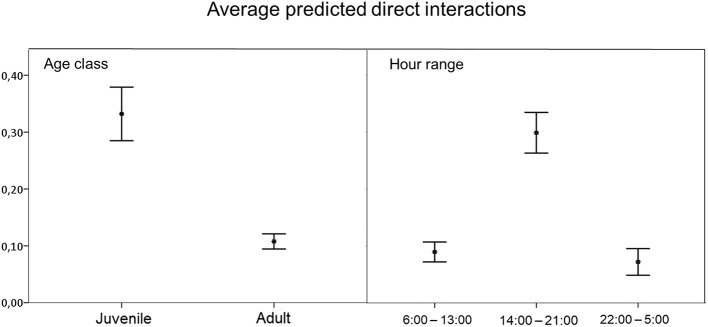
Average predicted number of direct interactions between free-ranging pigs and wild boar per animal observed based on statistically significant variables in the best-fit model. Error bars show the 95% confidence interval.

We observed 0.37 direct interactions per day (SD = 1.31; *n* = 140). The model to explain direct interaction between free-ranging pig and wild boar contained season, hour range, age, water source and pastureland as variables (Table 3). Direct interaction rate was positively associated with the hour range from 14:00 to 21:00 h, and negatively associated with adult animals (Figure 4), in other words, interactions occurred mainly among juveniles.

**Table 3:** Results of the best-fitting generalized linear mixed model (negative binomial distribution and log link function) to predict the rate of direct interaction between free-ranging pigs and wild boar.

		**Estimate**	**Std. error**	***Z* value**	***P*-value**
(intercept)		−16.90	38.46	−0.44	ns
Season 2	Summer	−0.28	1.83	−0.15	ns
Hour range 2	14–21 h	1.00	0.31	3.22	**
Hour range 3	22–5 h	0.67	0.44	1.51	ns
Age 2	Adult	−0.93	0.23	−4.04	***
Water source 2	Presence	1.26	2.66	0.47	ns
Pastureland 2	Presence	12.48	38.46	0.32	ns

P-values: p > 0.1 “ns”; p < 0.05 “^*^”; p <0.01 “^**^”; p < 0.001 “^***^”.

*Coefficients are relative to Season 1 (Spring), Hour range 1 (6–13 h), Age 1 (Juvenile), Water source 1 (Absence), Pastureland 1 (Absence)*.

### Indirect Interaction Rates

Our literature search for ASFV survival and infectious times in the environment identified 34 publications, but none reported survival times in the environment under field conditions. Therefore, we considered to define two CTWs based on the latest studies on survival time in excretions (feces and urine): a long CTW based on one estimate of survival time (42), corresponding to 7 days in spring (12°C) and 5 days in summer (21°C); and a short CTW based on the empirically short time window of 1 day for ASFV transmissibility (43).

Based on the short CTW, our results indicated 1.31 indirect interactions per day (SD = 6.64; *n* = 489). The corresponding model to explain indirect interactions contained season, activity, water source and pastureland as variables (Table 4). Indirect interaction rate based on short CTW was positively associated with movement (Figure 4).

**Table 4:** Results of the best-fitting generalized linear mixed model (negative binomial distribution and log link function) to predict the rate of indirect interaction between free-ranging pigs and wild boar assuming a short critical time window of 1 day for transmissibility of ASFV.

		**Estimate**	**Std. Error**	***Z* value**	***P*-value**
(intercept)		−2.62	0.80	−3.27	**
Season 2	Summer	0.11	0.46	0.24	ns
Activity 2	Moving	0.61	0.16	3.88	***
Water source 2	Presence	0.47	0.64	0.73	ns
Pastureland 2	Presence	−0.60	0.64	−0.93	ns

P-values: p > 0.1 “ns”; p < 0.01 “^**^”; p < 0.001 “^***^”.

*Coefficients are relative to Season 1 (Spring), Activity 1 (Other: drinking, pasturing, inspecting, resting, washing), Water source 1 (Absence), Pastureland 1 (Absence)*.

**Figure 5:**
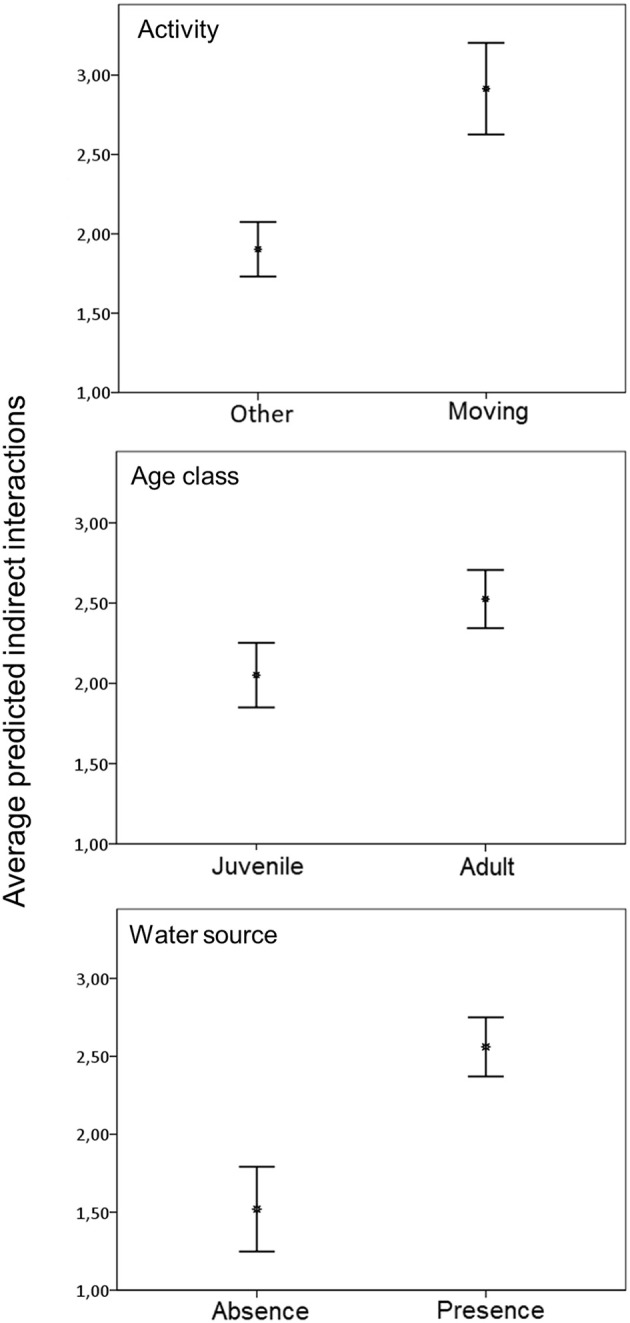
Average predicted number of indirect interactions between free-ranging pigs and wild boar per animal observed assuming a long critical time window of 7 days in spring and 5 days in summer for transmissibility of ASFV, based on statistically significant variables in the best-fit model. Error bars show the 95% confidence interval.

Based on the long CTW, our results indicated 6.47 indirect interactions per day (SD = 26.21; *n* = 2418). In this case, the corresponding model to predict indirect interactions contained season, direction of the interaction, age, activity, and water source as variables. Also, the final model identified the interaction between season and direction as significantly associated with indirect interaction rate (Table 5). Indirect interaction rate based on long CTW was also positively associated with movement. These indirect interactions usually occurred in the presence of a water source, and they involved adults more often than juveniles (Figure 5). In the summer, indirect interactions occurred more often in the direction of wild boar followed by free-ranging pig than in the opposite direction.

**Table 5:** Results of the best-fitting generalized lineal mixed model (negative binomial distribution and log link function) to predict the rate of indirect interaction between free-ranging pigs and wild boar assuming a long critical time window of 7 days in spring and 5 days in summer for transmissibility of ASFV.

		**Estimate**	**Std. Error**	**Z value**	***P*-value**
(intercept)		−1.78	1.03	−1.73	.
Season 2	Summer	−0.27	0.35	−0.77	ns
Direction 2	Free-ranging pig followed by wild boar	0.45	0.28	1.65	.
Age 2	Adult	0.23	0.10	2.39	*
Activity 2	Moving	0.28	0.09	3.01	**
Water source 2	Presence	0.76	0.38	1.99	*
Season 2: Direction 2	Summer: Free-ranging pig followed by wild boar	−0.87	0.29	−2.96	**

P-values: p > 0.1 “ns”; p < 0.1 “.”; p < 0.05 “^*^”; p < 0.01 “^**^”.

*Coefficients are relative to Season 1 (Spring), Direction 1 (Wild boar followed by free-ranging pig), Age 1 (Juvenile), Activity 1 (Other: drinking, pasturing, inspecting, resting, washing), Water source 1 (Absence)*.

## Discussion

This study provides the first evidence of interactions between free-ranging pigs and wild boar in the east-central part of Sardinia, and such interactions may help explain the endemicity of ASF. We observed higher rates of direct and indirect interactions between free-ranging pigs and wild boar than camera trapping studies on wildlife-domestic interface in other Mediterranean ecosystems (23, 32, 44), implying the relevance of this interaction in the epidemiology of ASF. Our study also confirms the usefulness of camera trapping for studying interspecific interactions more generally.

In our study, more animals were observed in the summer (*n* = 307) than in the spring (*n* = 229), and this increase in observations during summer was especially stronger for wild boar: 78% of all wild boar observations occurred in summer, compared to 63% of all free-ranging pig observations. The increase in observations during summer may be due to fewer food and water resources, reducing the home-range around natural resources (45). Reduction in home-range of free-ranging pigs may also occur if pig owners, to compensate for the shortage of natural resources during summer, supplement their animals' feed or even keep them on farms. Supplementing feed not only reduces the home-range size of free-ranging pigs but may attract wild boar. The increase in wild boar and free-ranging pig activity around natural resources in the summer may mean higher risk of contact with ASFV in the environment and therefore higher transmission risk.

Our rate of direct interactions in this area of Sardinia was considerably higher than the scarce or even undetectable rates reported in camera trapping studies of interactions between other wild ungulate and livestock species (23, 32, 44), and much higher than anecdotal direct interactions between wildlife and livestock in studies using other interaction-tracking methods (26, 28). Thus, our results provide a clear indication that wild boar and free-ranging pigs interact directly to a significant extent, highlighting the need to include this interface in epidemiological assessments of infectious swine pathogens, especially in extensive pig production systems.

Furthermore, our measured rates may underestimate direct interactions because we did not include the reproductive season from autumn to early winter, when most direct interactions occur between domestic pigs and wild boar (29). These reproductive interactions may have an important implication for understanding ASFV transmission, since the virus has been detected in semen and can be transmitted during mating (46). This lack of information on reproductive season may influence our finding that juveniles interacted directly more often than adults did, so this observation should be confirmed in further studies. The basic social organization of wild boar and free-ranging pigs is represented by male adults living singly and groups of females with juvenile offspring (47, 48). Males maintain greater distances with the rest of the adults than those maintained among female and juvenile groups (48), this behavior may explain the higher direct interaction rate observed in juveniles. Direct interactions between juveniles may have an impact on ASFV transmission and endemicity on the island, since young wild boar has previously been shown to be more likely to ASF seropositivity and virus positivity (49).

The frequency of direct interactions was significantly higher between 14:00 and 21:00 h (Figure 4), reflecting overlap in wild boar and free-ranging pig activity patterns (Figure 3). Overall, free-ranging pigs showed diurnal activity, while wild boar showed primarily nocturnal activity with sporadic diurnal activity, consistent with previous work in south central Spain (23). Domestic pigs on extensive or semi-extensive farms also show diurnal activity (23), so they may easily come into contact with free-ranging pigs in the absence of preventive measures, such as fencing fields where animals range free (50).

Our indirect interaction rate may also underestimate reality, since we had to define these interactions based on ASFV survival times in feces and urine because of a lack of studies on virus survival time in the environment. Viruses are likely to survive in feces and urine for less time than in blood, where they can persist for up to 15 weeks (51). Interaction between wild boar and carcasses has been described to occur frequently (52), which contributes to ASFV transmission and might also occur among free-ranging pigs. However, we did not capture carcasses of wild boar or free-ranging pigs on cameras.

Most indirect interactions in our study involved animals in movement, suggesting that wild boar and free-ranging pigs do not share resting areas. Overall, indirect interactions were much more frequent near water sources. These findings are similar to those for interactions between other species in the Mediterranean basin (23, 32, 33, 53). Animal congregation around water sources is considered one of the most important factor for pathogen transmission between wildlife and livestock (28, 54). While ASFV survival time at natural water sources is unclear, infectious titers are considerably lower when the virus is transmitted in liquid than in feed (55). In addition, a recent study has shown the potential role of leeches to harbor ASFV, where the virus could remain active up to 140 days (56). Therefore, control measures should target water sources, as proposed for other infectious diseases (28, 50).

Another additional factor to take account when modeling direct and indirect interaction rates is the population density or abundance. Theoretically, we expect an increasing in contact rates (higher risk of pathogen transmission) with higher density but saturates upon reaching a threshold of population density (57). However, in the present study, we could not consider this factor due to the lack of availability of abundance and density data of wild boar and free-ranging pig populations at suitable spatial scale, but it would be greatly recommended for further studies.

## Conclusion

Our results provide the first conservative estimates of interactions between free-ranging pigs and wild boar interactions in Sardinia. The likelihood that our data underestimate actual interactions further underscores the importance of this interface for understanding ASFV transmission. Such interactions may therefore quite reasonably account for the longstanding ASF endemicity on the island of Sardinia, and they support the need to eliminate free-ranging pig breeding practices. More broadly, we consider the control of free-ranging pigs as an important measure against ASFV transmission, taking especial attention during summer, at water sources and between 14:00 and 21:00 h. The findings of this study may help to model the spread of ASFV in the context of the domestic-wild swine interface, but it should be assessed in other epidemiological scenarios. Finally, we conclude that analysis of interactions between free-ranging pigs and wild boar has great potential for guiding effective prevention policies and evaluating disease management.

## Data Availability Statement

All datasets generated for this study are included in the article/Supplementary Material.

## Author Contributions

JS-V, AP, and JV designed the study. JS-V, AP, JV, DD, MC, and JB carried out the field work. EC-F and CJ collected the data. EC-F and JB performed the analyses. EC-F, JS-V, CJ, and JB wrote the manuscript. All authors revised the manuscript and approved the final version.

### Conflict of Interest

The authors declare that the research was conducted in the absence of any commercial or financial relationships that could be construed as a potential conflict of interest.
