# Supplementary material for: Free-Ranging Pig and Wild Boar Interactions in an Endemic Area of African Swine Fever
Source: Front Vet Sci. 2019 Oct 30;6:376. doi: 10.3389/fvets.2019.00376 (PMC6831522; doi:10.3389/fvets.2019.00376)
Supplement: Supplementary file 1 [file Table_1.pdf]

Supplementary Material 2

Image processing

Interactions between free-ranging pig and wild boar were determined as follows:

1. Load the data information (named: data) as csv file in a data base MySQL
2. Two image processing were carried out based on the direction of the interactions (free-ranging pig->wild boar, wild boar->free-ranging pig)
3. Start date and time was recorded as **date1** for the first subspecies observed and as **date 0** for the next subspecies observed.

Direction: free-ranging pig->wild boar

4. Critical time windows (CTW) were obtained from the start date and time of each free-ranging pig observation as follow:

   a. Short CTW: start date and time + 1 day -> **date2**
   b. CTW: start date and time + (7 days (in spring) or 5 days (in summer) -> **date3**

```
if($row['free-ranging pig']==1){
        $date1=$row['date'];
        $date2 = strtotime ('+1 day',strtotime($row['date']." ".$row['hour']));
        if ($row['season']==1){
        $date3 = strtotime ('+7 day',strtotime($row['date']." ".$row['hour']));
} else {
        $date3 = strtotime ('+5 day',strtotime($row['date']." ".$row['hour']));
        }
}
```

5. Interactions were determined from wild boar observations of the same camera trapping within the CTW obtained in the step before.

//Wild boar observations from the same camera trapping

```
SELECT * FROM data WHERE IDcamera='".$row['IDcamera']."' AND wildboar=1
```

//Direct interactions. The start date and time of each wild boar is named *date0*

```
if ($date0 == $date1){
        $counter1++;
}
```

//Indirect interactions for short CTW

```
if ($date0 >= $date1 && $date0<=$date2){
        $counter2++;
}
```

//Indirect interactions for CTW

```
if ($date0 >= $date1 && $date0<=$date3){
        $counter3++;
}

$counter=$counter1+$counter2+$counter3;
```

//Counter = number of interactions for each free-ranging pig observation
//Counter1 = direct interactions
//Counter2 = indirect interactions for short CTW
//Counter3= indirect interactions for CTW

Direction: wild boar->free-ranging pig

4. Critical time windows (CTW) were obtained from the start date and time of each wild boar observation as follow:

    a. Short CTW: start date and time + 1 day -> **date2**
    b. CTW: start date and time + (7 days (in spring) or 5 days (in summer) -> **date3**

```
if($row['wildboar']==1){
        $date1=$row['date'];
        $date2 = strtotime ('+1 day',strtotime($row['date']." ".$row['hour']));
        if ($row['season']==1){
        $date3 = strtotime ('+7 day',strtotime($row['date']." ".$row['hour']));
} else {
        $date3 = strtotime ('+5 day',strtotime($row['date']." ".$row['hour']));
        }
}
```

5. Interactions were determined from free-ranging pig observations of the same camera trapping within the CTW obtained in the step before.

//Free-ranging pig observations from the same camera trapping

```
SELECT * FROM data WHERE IDcamera='".$row['IDcamera']."' AND free-ranging pig=1
```

//Direct interactions. The start date and time of each free-ranging pig is named *date0*

```
if ($date0 == $date1){
        $counter1++;
}
```

//Indirect interactions for short CTW

```
if ($date0 >= $date1 && $date0<=$date2){
        $counter2++;
}
```

```
//Indirect interactions for CTW

if ($date0 >= $date1 && $date0<=$date3){
        $counter3++;
}

$counter=$counter1+$counter2+$counter3;

//Counter = number of interactions for each free-ranging pig observation
//Counter1 = direct interactions
//Counter2 = indirect interactions for short CTW
//Counter3= indirect interactions for CTW
```
