# Supplementary figures and images for: Free-Ranging Pig and Wild Boar Interactions in an Endemic Area of African Swine Fever
Source: Front Vet Sci. 2019 Oct 30;6:376. doi: 10.3389/fvets.2019.00376 (PMC6831522; doi:10.3389/fvets.2019.00376)

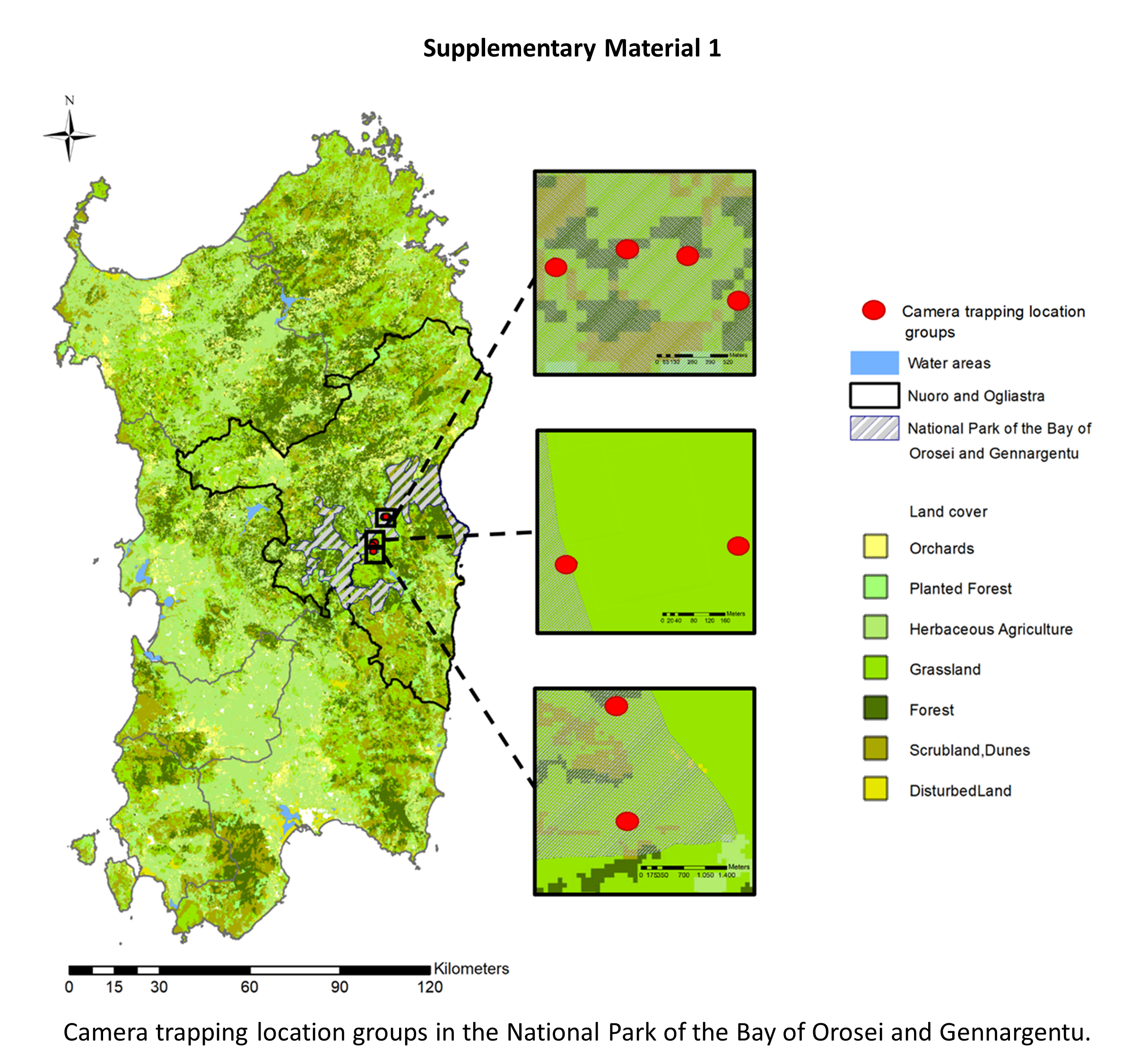

Supplement: Supplementary file 2 [file Image_1.tif]
